# Willingness to Use and Pay for Telemedicine and Teleconsultation Across Five Clinical Domains in South Korea: Cross-Sectional Survey

**DOI:** 10.2196/65304

**Published:** 2025-06-16

**Authors:** Hajae Jeon, Jeahyung Lee, Jieun Jang, Mingee Choi, Junbok Lee, Jaeyong Shin

**Affiliations:** 1 Department of Public Health Graduate School Yonsei University Seoul Republic of Korea; 2 Institute for Innovation in Digital Healthcare Yonsei University Seoul Republic of Korea; 3 Department of Economics College of Commerce and Economics Yonsei University Seoul Republic of Korea; 4 Department of Preventive Medicine Dongguk University College of Medicine Gyeongsangbuk-do Republic of Korea; 5 Department of Preventive Medicine Yonsei University College of Medicine Seoul Republic of Korea; 6 Institute of Health Services Research Yonsei University Seoul Republic of Korea

**Keywords:** digital health, health technology, patient preferences, telehealth

## Abstract

**Background:**

The COVID-19 pandemic accelerated global telehealth adoption, prompting the South Korean government to temporarily legalize telemedicine in 2020 and subsequently launch a pilot program in 2023. As South Korea transitions to a postpandemic digital health environment, understanding the factors associated with willingness to use (WTU) and willingness to pay (WTP) for telemedicine and teleconsultation is essential for informing effective policy and service design. However, few studies have explored how preferences vary across clinical domains or user groups.

**Objective:**

This study examined the factors that influence WTU and WTP for telemedicine and teleconsultation across 5 clinical domains: dermatological, psychiatric, musculoskeletal, internal medicine, and cancer disorders.

**Methods:**

A cross-sectional survey was conducted among 552 participants aged 19-69 years in South Korea, selected through stratified sampling. Multiple logistic regression analysis was used to examine WTU and WTP, considering sociodemographic factors and previous telemedicine experience.

**Results:**

Participants’ age, residence, and previous telemedicine experience significantly influenced their WTU and WTP for telemedicine services. WTP increased with age for both telemedicine (*P*-for-trend=.02) and teleconsultation (*P*-for-trend=.001). Noncapital residents showed significantly higher WTU for teleconsultation than capital area residents (odds ratio [OR] 1.48, 90% CI 1.03-2.12; *P*=.07). Participants with previous telemedicine experience showed higher WTU for telemedicine (OR 4.07, 90% CI 1.84-9.04; *P*=.004) and teleconsultation (OR 2.21, 90% CI 1.21-4.06; *P*=.03), and higher WTP for telemedicine (OR 2.89, 90% CI 1.84-4.54; *P*<.001) and teleconsultation (OR 2.76, 90% CI 1.77-4.30; *P*<.001). WTU and WTP varied by clinical domain: psychiatric care showed the highest WTU (64.5%) and WTP (27.0%) for telemedicine, while cancer disorders showed higher WTU (48.6%) and WTP (24.8%) for teleconsultation than for telemedicine.

**Conclusions:**

WTU and WTP for telemedicine and teleconsultation differ substantially depending on service type, clinical domain, and user characteristics. These findings highlight the importance of considering prior telemedicine experience, regional access disparities, and condition-specific care needs when designing digital health strategies. Accordingly, flexible, user-centered telehealth policies are needed to support service accessibility and equitable implementation in the post–COVID-19 era. The insights from this study can serve as a practical foundation for developing inclusive digital health systems in countries undergoing similar transitions.

## Introduction

Telemedicine involves using information or communication technology to deliver health care services, replacing traditional face-to-face physician-patient encounters [1]. Its benefits include improved access, reduced costs from fewer emergency visits, and enhanced patient satisfaction [2-4]. The COVID-19 pandemic significantly accelerated the global adoption of telemedicine and digital health technologies [5-7].

**Figure 1:**
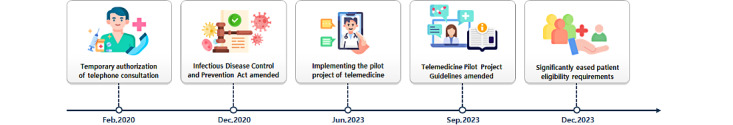
The changes in South Korea’s telemedicine policy during the post–COVID-19 period.

Before the pandemic, however, telemedicine had been prohibited in South Korea due to legal and regulatory barriers. Nevertheless, the COVID-19 crisis created an urgent need for non-face-to-face care, prompting a series of policy changes. In response, the Korean government temporarily permitted telemedicine for the first time in February 2020 [8-10]. Following the end of the pandemic in June 2023, the Korean government launched a “Telemedicine Pilot Project” to introduce telemedicine in earnest, and in December 2023, it announced an amendment to the project to broaden telemedicine’s eligibility criteria [11] (Figure 1 and Table S1 in Multimedia Appendix 1).

Alongside the expansion of telemedicine, the pandemic also sparked broader interest in digital health care innovations, such as remote monitoring and (physician-physician) teleconsultation [12]. Teleconsultation entails “a doctor at a remote location advising a distant medical practitioner on the medical process with knowledge or technology” [13,14]. In Korea, it is generally understood as a service wherein a medical practitioner at a large hospital and one at a local hospital communicate remotely and treat patients together. As the current medical law allows for the implementation of teleconsultation in Korea, teleconsultation has recently gained more attention because it reduces unnecessary transportation and enhances medical services [14].

As telemedicine and teleconsultation have gained traction in the postpandemic era, understanding the factors that influence user willingness to use and pay (WTU and WTP) for such services has become crucial for informing sustainable policy development and service design [15]. In addition, user perspectives on these services may have shifted during the COVID-19 pandemic, as many individuals experienced telehealth for the first time or developed new attitudes toward remote care. However, there remains a lack of comprehensive evidence on the determinants of WTU and WTP, particularly within the context of South Korea’s unique regulatory environment and recent policy transitions. Therefore, new research is needed to prepare for telehealth in the post-COVID-19 era and examine the utilization intentions and WTP of users who experienced limited access to telemedicine during COVID-19.

Moreover, patients’ WTU and WTP for telemedicine and teleconsultation may vary substantially across clinical domains, reflecting differences in disease characteristics, care needs, and perceived utility of remote services. To our knowledge, few studies have analyzed and reported population differences in telemedicine and teleconsultation services based on clinical domain services.

This study aimed to address this gap by investigating WTU and WTP for telemedicine and teleconsultation across 5 clinical domains in a national sample. In addition, we sought to identify the relevant determinants of WTU and WTP and explore how these outcomes varied across user groups and clinical domains.

## Methods

### Participants

We conducted a nationwide web-based mobile survey of people aged 19-69 years. Participants were recruited via email from a research company’s large web-based panel of approximately 70,000 registered panelists. To ensure national representativeness, we used stratified sampling based on 3 variables: gender (male or female), age (in five 10-year bands: 19-29, 30-39, 40-49, 50-59, and 60-69), and residential region (categorized into 7 administrative areas). Participants were proportionally allocated to each stratum to reflect the demographic composition of the South Korean population. As of November 2023, 666 participants had completed the survey.

### Design and Procedure

We provided detailed text- and image-based explanations to participants to help avoid confusion related to the concepts of telemedicine and teleconsultation. Telemedicine was defined as “a medical service that allows patients to consult a doctor by using a smartphone or computer without physically going to a hospital.”

Teleconsultation, however, was explained as “a service in which a patient visits a nearby hospital instead of going far away to a university hospital, such as in a metropolitan area, and a doctor from a large hospital and one from a nearby hospital connect remotely through a computer to treat the patient together.” Following these explanations, the participants were presented with 2 questions to verify their understanding of the distinction. Participants who answered either question incorrectly were excluded from the final sample; this resulted in the selection of 552 participants from the initial 666.

The participants responded to the WTU and WTP questions under the assumption of a complete implementation of telemedicine and teleconsultation in Korea. In addition to the overall perspective, both WTU and WTP were assessed for each medical service department. The detailed departments were divided into 5 categories, which were guided by criteria from prior studies and departments conducted for pilot studies and telemedicine during the COVID-19 period in Korea. Participants were presented with examples of the clinical domains for each service. Specifically, dermatological disorders, acute and chronic skin conditions, dermatological consultations, psoriasis, atopic dermatitis, and various types of wounds and rashes were identified. Psychiatric disorders, anxiety disorders, depressive disorders, adjustment disorders, medication management, individual therapy, posttraumatic stress disorder, and substance abuse treatments were outlined. Internal medical disorders included diabetes, heart failure, hyperlipidemia, myocardial infarction, and thyroid disorders. Musculoskeletal disorders included chronic pain (back, shoulders, knees, hands, ankles, neck, etc), arthritis, fractures, rehabilitation therapy following musculoskeletal surgery, and chronic degenerative joint disorders. Finally, cancer disorders included thyroid, lung, colorectal, stomach, liver, pancreatic, gallbladder, other biliary tract, and renal cancers. To obtain a measure of WTU, the survey included the following question: “Would you be willing to utilize telemedicine or teleconsultation services once they are fully implemented?” For WTP, a comparison with traditional medical services was included in the questionnaire to obtain a more accurate measure. Specifically, the survey included the following question: “Would you be willing to pay extra for the platform to access telemedicine or teleconsultation?” Subsequently, participants received the following clarification: “This question pertains to the additional amount of money you would pay to access telemedicine or teleconsultation, assuming that the relevant cost of care is equivalent to that of in-person care.”

### Sociodemographics and Health Status

Participants provided information about their age, gender, and residence. They also shared their experiences with telemedicine, their subjective health status (whether they smoked or consumed alcohol), their subjective perception of distance to a hospital, and any chronic conditions (eg, hypertension and diabetes), mental illness (eg, depression), or cancer.

In Article 2 of the Seoul Capital Area Development Act, “residence” is defined as living either within the Seoul capital area or in the vicinity of the capital area [16]. More than half of the Republic of Korea’s population resides in the Seoul capital area, which is characterized by a high density of comprehensive hospitals relative to its area [17,18] (Figure S1 in Multimedia Appendix 1).

### Dependent Variable, WTU, and WTP Questions

#### Willingness to Use

WTU was measured on a 4-point scale. Participant responses indicating “somewhat willing” or “highly willing” were classified under “willing to use.” A 4-point scale was also used to capture participants’ responses regarding each of the 5 medical department services when telemedicine and teleconsultation were offered in each of the 5 clinical domains. “Highly willing” and “somewhat willing” responses were classified into the “willing to use” group. Responses of “not at all” and “not very much” were placed in the “reluctant” group.

#### Willingness to Pay

Participants were asked to respond “yes” or “no” regarding their WTP an additional fee for the platform to use telemedicine and teleconsultation services. In addition, for each of the 5 clinical domains, participants were asked whether they would be willing to use telemedicine and teleconsultation services if they cost more than traditional medical services (in-person or offline).

### Statistical Analysis

We conducted multiple logistic regression analyses to investigate the relationship between WTU and WTP for telemedicine and teleconsultation; our analysis accounted for demographic characteristics as well as participants’ prior experiences of telemedicine and health status. Effect sizes were reported as odds ratios (ORs) with 90% CIs. In addition, the WTU and WTP were examined across 5 clinical domains: dermatological, psychiatric, musculoskeletal, internal medicine, and cancer disorders. A 2-sided significance level of α=.10 was applied to reflect the exploratory nature of the study and to identify potentially meaningful associations. All statistical analyses were performed using SAS version 9.4 (SAS Institute Inc.).

### Ethics Approval

This study was approved by the Institutional Review Board (IRB) of the Yonsei University Health System, Severance Hospital (IRB number 4-2023-1333). All participants received a detailed explanation of the study’s purpose, procedures, potential risks, and benefits through an online platform, and provided informed consent electronically prior to participation. Participants were assured that their privacy and confidentiality would be strictly protected, and all data were anonymized prior to analysis. No personal identifying information was collected or stored. Participants received a compensation of 1500 South Korean won (US $1.10) for completing the survey.

## Results

### Participants’ General Characteristics

Table S2 in Multimedia Appendix 1 presents the participants’ demographic distribution. Our analysis revealed variations in the WTU and WTP for telemedicine and teleconsultations across different demographic groups.

### WTU and WTP for Telemedicine and Teleconsultations

Tables 1 and 2 present the results of the logistic regression analysis of the WTU and WTP for telemedicine services. As age increased, there was a tendency toward increased WTU and WTP for telemedicine and teleconsultation, compared with the tendency in the 19-29 years age group. As age increased, there was a significant increase in WTP for telemedicine (*P*-for-trend=.02).

**Table 1:** Results of factors associated with willingness to use and pay for telemedicine.

Variables		Willing to use		Willing to pay			
		OR^a^ (90% CI)	*P* value	OR (90% CI)			*P* value
**Sex**							
	Female	Ref^b^	—^c^		Ref	—	
	Male	1.07 (0.70-1.63)	.80		0.88 (0.62-1.25)	.55	
**Age group (years)**							
	19-29	Ref	—		Ref	—	
	30-39	0.62 (0.32-1.20)	.60		0.92 (0.53-1.58)	.06	
	40-49	0.65 (0.34-1.26)	.74		1.78 (1.05-3.00)	.12	
	50-59	0.47 (0.25-0.89)	.049		1.22 (0.73-2.04)	.60	
	60+	0.89 (0.44-1.81)	.36		2.13 (1.24-3.67)	.02	
**Residence**							
	Seoul Capital Area	Ref	—		Ref	—	
	Noncapital	0.96 (0.66-1.39)	.84		0.92 (0.68-1.25)	.67	
**Alcohol use**							
	No	Ref	—		Ref	—	
	Yes	1.29 (0.78-2.13)	.41		0.98 (0.65-1.48)	.95	
**Smoking**							
	Nonsmoker	Ref	—		Ref	—	
	Ex-smoker	1.96 (1.13-3.43)	.03		0.93 (0.61-1.42)	.60	
	Current smoker	1.01 (0.63-1.63)	.22		1.10 (0.73-1.65)	.55	
**Distance to health care (on foot)**							
	Less than 5 min	Ref	—		Ref	—	
	5 to 9 min	0.63 (0.31-1.27)	.80		0.74 (0.43-1.25)	.67	
	10 to 29 min	0.49 (0.23-1.04)	.13		0.69 (0.39-1.22)	.40	
	30 min or more	0.63 (0.24-1.69)	.88		0.77 (0.35-1.68)	.92	
**Subjective distance to health care**							
	Very close	Ref	—		Ref	—	
	Close	1.55 (0.88-2.73)	.16		1.01 (0.64-1.60)	.38	
	Far	1.12 (0.49-2.59)	.80		0.68 (0.33-1.41)	.30	
**Experiences of telemedicine**							
	No	Ref	—		Ref	—	
	Yes	4.07 (1.84-9.04)	.004		2.89 (1.84-4.54)	<.001	
**Self-rated health**							
	Bad	Ref	—		Ref	—	
	Good	1.18 (0.77-1.80)	.53		1.64 (1.17-2.31)	.02	
**Experiences of chronic disease**							
	No	Ref	—		Ref	—	
	Yes	1.06 (0.71-1.58)	.82		1.92 (1.38-2.68)	.001	
**Experiences of mental disease**							
	No	Ref	—		Ref	—	
	Yes	0.90 (0.52-1.54)	.74		1.19 (0.77-1.86)	.51	

^a^OR: odds ratio.

^b^Reference values.

^c^Not applicable.

**Figure 2:**
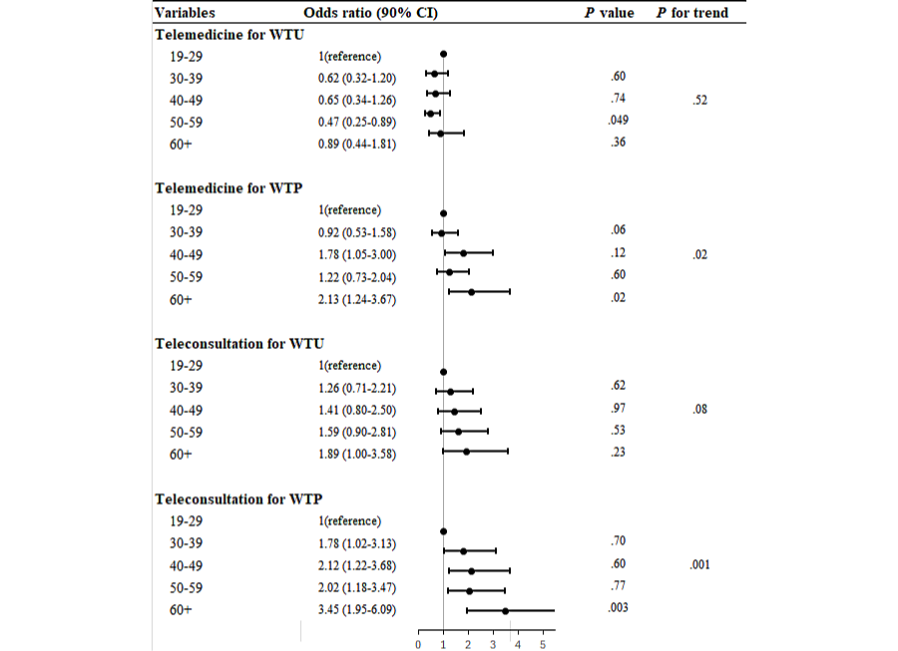
Willingness to use and willingness to pay for telemedicine and teleconsultation across age groups. WTP: willingness to pay; WTU: willingness to use.

In addition, both WTU and WTP for teleconsultation showed an upward trend as age increased with a 10% significance level (WTU: *P*-for-trend=.08; WTP: *P*-for-trend=.001) (Figure 2).

Noncapital cities’ residents exhibited a significantly higher WTU for teleconsultation compared to Seoul capital area residents, with a 10% significance level (OR 1.48, 90% CI 1.03-2.12; *P*=.07), while no regional differences were observed in this regard for telemedicine.

Individuals with prior experiences with telemedicine services exhibited significantly greater WTU and WTP for telemedicine compared to those without such experiences (WTU: OR 4.07, 90% CI 1.84-9.04; *P*=.004; WTP: OR 2.89, 90% CI 1.84-4.54; *P*<.001).

Those with prior telemedicine experience showed higher WTU and WTP for teleconsultation (WTU: OR 2.21, 90% CI 1.21-4.06; *P*=.03; WTP: OR 2.76, 90% CI 1.77-4.30; *P*<.001).

Furthermore, individuals with better self-rated health showed significantly higher WTP for telemedicine (OR 1.64, 90% CI 1.17-2.31; *P*=.02) as well as higher WTU (OR 1.63, 90% CI 1.08-2.46; *P*=.05) and WTP (OR 1.57, 90% CI 1.11-2.22; *P*=.03) for teleconsultation compared to those with poorer health.

Individuals who had experiences with chronic conditions demonstrated significantly higher WTP for both telemedicine (OR 1.92, 90% CI 1.38-2.68; *P*=.001) and teleconsultation (OR 1.77, 90% CI 1.27-2.48; *P*=.005) compared to those without such diagnoses.

**Table 2:** Results of factors associated with willingness to use and pay for teleconsultation.

Variable		Willing to use		Willing to pay			
		OR^a^ (90% CI)	*P* value	OR (90% CI)			*P* value
**Sex**							
	Female	Ref^b^	—^c^		Ref	—	
	Male	0.67 (0.42-0.96)	.07		0.90 (0.63-1.28)	.62	
**Age group (years)**							
	19-29	Ref	—		Ref	—	
	30-39	1.26 (0.71-2.21)	.62		1.78 (1.02-3.13)	.70	
	40-49	1.41 (0.80-2.50)	.97		2.12 (1.22-3.68)	.60	
	50-59	1.59 (0.90-2.81)	.53		2.02 (1.18-3.47)	.77	
	60+	1.89 (1.00-3.58)	.23		3.47 (1.95-6.09)	.003	
**Residence**							
	Seoul Capital Area	Ref	—		Ref	—	
	Noncapital	1.48 (1.03-2.12)	.07		1.08 (0.79-1.46)	.69	
**Alcohol use**							
	No	Ref	—		Ref	—	
	Yes	1.32 (0.81-2.15)	.34		1.09 (0.72-1.65)	.73	
**Smoking**							
	Nonsmoker	Ref	—		Ref	—	
	Ex-smoker	2.40 (1.40-4.11)	.03		0.95 (0.62-1.44)	.70	
	Current smoker	1.59 (1.00-2.52)	.93		1.07 (0.71-1.61)	.68	
**Distance to healthcare (on foot)**							
	Less than 5 min	Ref	—		Ref	—	
	5 to 9 min	0.73 (0.38-1.41)	.83		0.82 (0.49-1.40)	.81	
	10 to 29 min	0.60 (0.30-1.22)	.23		0.61 (0.34-1.08)	.04	
	30 min or more	0.75 (0.30-1.90)	.98		1.07 (0.49-2.36)	.43	
**Subjective distance to health care**							
	Very close	Ref	—		Ref	—	
	Close	0.97 (0.55-1.69)	.92		0.93 (0.59-1.47)	.35	
	Far	0.98 (0.43-2.25)	1.00		0.55 (0.27-1.16)	.15	
**Experiences of telemedicine**							
	No	Ref	—		Ref	—	
	Yes	2.21 (1.21-4.06)	.03		2.76 (1.77-4.30)	<.001	
**Self-rated health**							
	Bad	Ref	—		Ref	—	
	Good	1.63 (1.08-2.46)	.05		1.57 (1.11-2.22)	.03	
**Experiences of chronic disease**							
	No	Ref	—		Ref	—	
	Yes	1.43 (0.97-2.11)	.13		1.77 (1.27-2.48)	.005	
**Experiences of mental disease**							
	No	Ref	—		Ref	—	
	Yes	0.88 (0.52-1.48)	.69		1.18 (0.76-1.85)	.53	

^a^OR: odds ratio.

^b^Reference values.

^c^Not applicable.

### WTU and WTP for Telemedicine and Teleconsultations in Five Clinical Domains

We investigated WTU and WTP for telemedicine services in 5 clinical domains (Table 3 and Tables S3-S7 in Multimedia Appendix 1).

Table 3 presents WTU and WTP for telemedicine and teleconsultation services across the different clinical domains. The highest WTU (64.5%) and WTP (27.0%) values for telemedicine were observed among patients with psychiatric disorders. Conversely, telemedicine for cancer disorders received lower levels of acceptance, with WTU and WTP percentages of 27.9% and 15.0%, respectively. Contrastingly, teleconsultations received markedly higher levels of acceptance with regard to cancer disorders, with a WTU of 48.6% and a WTP of 24.8%.

Individuals residing in noncapital areas exhibited a higher WTU tendency for telemedicine concerning musculoskeletal disorders and internal medicine disorders compared to those residing in capital areas, with a 10% significance level (musculoskeletal: OR 1.41, 90% CI 1.05-1.91; *P*=.06; internal medicine: OR 1.44, 90% CI 1.06-1.94; *P*=.047) (Tables S3 and S4 in Multimedia Appendix 1). Moreover, residents of noncapital areas demonstrated a higher WTU for teleconsultation regarding dermatological disorders and cancer disorders compared to their counterparts in capital areas, with a 10% significance level (dermatological: OR 1.41, 90% CI 1.04-1.91; *P*=.07; cancer: OR 1.63, 90% CI 1.21-2.19; *P*=.007) (Tables S5 and S6 in Multimedia Appendix 1). In addition, residents in noncapital areas displayed a greater WTP for teleconsultation regarding musculoskeletal disorders, internal medicine disorders, dermatological disorders, and cancer disorders (musculoskeletal: OR 1.58, 90% CI 1.12-2.23; *P*=.03; internal medicine: OR 1.43, 90% CI 1.02-2.01; *P*=.08; dermatological: OR 1.47, 90% CI 1.04-2.09; *P*=.07; cancer: OR 1.60, 90% CI 1.14-2.25; *P*=.02) (Tables S3-S6 in Multimedia Appendix 1).

Table S6 in Multimedia Appendix 1 shows that individuals with prior experiences with telemedicine services exhibited a higher WTU for cancer disorders (OR 1.64, 90% CI 1.04-2.59; *P*=.08), and Tables S3-S7 in Multimedia Appendix 1 demonstrated a higher WTP for telemedicine across all categories, including musculoskeletal disorders (OR 2.27, 90% CI 1.38-3.75; *P*=.007), internal medicine disorders (OR 2.71, 90% CI 1.69-4.34; *P*=.001), dermatological disorders (OR 2.95, 90% CI 1.86-4.69; *P*<.001), cancer disorders (OR 1.81, 90% CI 1.06-3.11; *P*=.07), and psychiatric disorders (OR 2.34, 90% CI 1.51-3.61; *P*=.001).

**Table 3:** WTU and WTP for telemedicine and teleconsultation in 5 clinical domains (N=552).

Category			Willing to use		Willing to pay
			Value, n (%)		Value, n (%)
**Telemedicine**					
	Dermatological	331 (60.0)		106 (19.2)	
	Psychiatric	356 (64.5)		149 (27.0)	
	Musculoskeletal	270 (48.9)		93 (16.8)	
	Internal medicine	303 (54.9)		109 (19.7)	
	Cancer	154 (27.9)		83 (15.0)	
**Teleconsultation**					
	Dermatological	349 (63.2)		126 (22.8)	
	Psychiatric	334 (60.5)		149 (27.0)	
	Musculoskeletal	318 (57.6)		138 (25.0)	
	Internal medicine	348 (63.0)		141 (25.5)	
	Cancer	268 (48.6)		137 (24.8)	

Similarly, a higher WTU was observed for teleconsultation specifically for dermatological disorders (OR 1.84, 90% CI 1.15-2.94; *P*=.03) along with an increased WTP for teleconsultation across all mentioned categories: musculoskeletal disorders (OR 2.17, 90% CI 1.38-3.41; *P*=.005), internal medicine disorders (OR 1.68, 90% CI 1.07-2.65; *P*=.06), dermatological disorders (OR 1.75, 90% CI 1.09-2.79; *P*=.05), cancer disorders (OR 1.87, 90% CI 1.19-2.95; *P*=.02), and psychiatric disorders (OR 1.86, 90% CI 1.19-2.88; *P*=.02) (Tables S3-S7 in Multimedia Appendix 1).

## Discussion

This study examined factors associated with WTU and WTP for telemedicine and teleconsultation across 5 clinical domains. Participants with previous experience using telemedicine showed consistently higher WTU and WTP for both service types. Age, health status, and regional factors were also associated with WTU and WTP in specific subgroups. Furthermore, WTU and WTP varied across clinical domains, suggesting that preferences may differ depending on the characteristics of each condition.

The older the age, the higher the WTP for telemedicine and the WTU and WTP for teleconsultation. However, those in their 20s showed the highest WTU for telemedicine. As many non-face-to-face services are mainly provided through apps, younger people who are familiar with such technologies have a higher WTU, given the convenience of using such services.

We obtained slightly different results for region-related factors with regard to telemedicine and teleconsultation. For teleconsultation, residents of noncapital areas showed a higher WTU than those in the capital area, whereas there was no significant difference based on capital or noncapital residence for telemedicine. For both capital and noncapital areas, more than 80% of the respondents were willing to use telemedicine. This might reflect convenience-related expectations such as not having to visit a hospital in person [19,20]. Contrastingly, regarding teleconsultation, patients in noncapital areas are more likely to use teleconsultation given that it is relatively difficult for them to visit large hospitals in person.

Experience with telemedicine services was a factor that increased WTU and WTP for both telemedicine and teleconsultation. When analyzed for each specific condition, WTP tended to be higher among those with prior experience across all disorders. This is likely owing to positive experiences with telemedicine services (eg, convenience and efficiency) [21-23], which make it easier for patients to receive care. Qualitative studies involving individuals who were willing to use telemedicine or reported higher satisfaction with telemedicine services indicated that time and cost savings were key factors driving telemedicine acceptance [24-26]. Such findings align with our study, suggesting that the perceived convenience and financial benefits associated with prior telemedicine experience might help explain the higher WTU and WTP observed in our participants. Studies have also shown that experience with digital health care positively influences WTP and WTU for service [27]. It is expected that more experiences with telemedicine and teleconsultation will positively influence this field’s scalability [28].

Individual health status was also a factor that influenced WTU and WTP for telemedicine and teleconsultation. People with chronic conditions tended to have a higher WTP for telemedicine and teleconsultation. This finding is similar to previous research suggesting that people are more willing to pay for these services because they have medical problems [22]. In addition, people with higher self-rated health statuses had higher WTU and WTP for telemedicine and consultation, and former cigarette smokers tended to have higher WTU, which may support this argument.

Examining the highlights for each disorder, WTP and WTU for telemedicine were the highest for psychiatric disorders. It can be posited that psychiatric disorders often require longer counseling sessions and that individuals with such disorders may exhibit a heightened propensity for public reticence compared to those with other medical conditions. This preference could contribute significantly to the growing demand for telehealth services over in-person consultations.

Contrastingly, WTU (27.9%) and WTP (15.5%) for telemedicine for cancer were low, whereas WTU (48.6%) and WTP (24.8%) for teleconsultation in this domain were relatively high. Compared with WTU for teleconsultation and telemedicine in other specialties, there is a large discrepancy between consultation and telemedicine in the cancer domain. Cancer is a severe, complex disease, and most Korean patients prefer treatment at tertiary hospitals, which offer advanced expertise and specialized care [29]. Evidence suggests that patients treated at such institutions have better outcomes [30]. However, prolonged wait times at tertiary hospitals pose challenges [31], especially for those requiring urgent care. Teleconsultation helps address such issues by allowing local physicians to collaborate with specialists at tertiary hospitals. This approach improves access to expert care while reducing the burden of travel and delays. Noncapital residents, who reported a higher WTU (54.1%) for teleconsultation in cancer disorders, might particularly benefit from this system. In addition to accessibility issues, patients with cancer and their families face considerable psychological distress, making physician interaction a critical aspect of care [32,33]. While telemedicine limits in-person engagement, teleconsultation maintains face-to-face interactions with local physicians while incorporating specialist input. These combined advantages might explain the higher WTU and WTP for teleconsultation in cancer care.

Considering the characteristics of telemedicine and teleconsultation services, we assumed that the distance between the residence and the hospital would affect service use. Contrary to expectation, neither objective nor subjective distance affected WTP and WTU for telemedicine and teleconsultation, except that subjective distance affected WTP for telemedicine in the case of cancer. This could be related to Korea’s unique characteristics. Specifically, Korea has a national health insurance system that makes it easy for patients to visit hospitals, giving them good access to medical care [23].

However, this study had some limitations. First, participants were presented with hypothetical scenarios, and this prompted them to respond based on their imagined reactions to the given situations; therefore, their responses to analogous real-life situations may vary significantly. This limitation can be attributed to the current policy landscape in Korea, in which evidence for implementing remote healthcare is lacking. To address this limitation, detailed explanations of telemedicine services and examples specific to medical specialties were provided to concretize the hypothetical situations. In addition, participants who answered the comprehension questions incorrectly were excluded to ensure an accurate interpretation of these core concepts. While this decision aimed to enhance internal validity, it may have inadvertently excluded individuals with lower digital or health literacy, thereby limiting the generalizability of the findings. As telemedicine and teleconsultation become more established in clinical practice and public understanding improves, future studies may be able to assess these services without the need for conceptual screening, thereby enhancing generalizability to broader populations.

Second, the discrepancies in health care insurance frameworks among countries may result in variations in both WTU and WTP, potentially deviating from this study’s outcomes. Furthermore, attention is required when directly interpreting the WTP presented in this study, as it reflects the participants’ willingness to incur additional expenses compared to traditional in-person consultations. In addition, we measured WTP using a binary (yes or no) response format, which does not capture the extent to which individuals are willing to pay. This approach was deliberately selected to provide a clear, interpretable comparison of WTP and WTU across 5 clinical domains rather than to quantify the exact monetary value participants were willing to pay. Although this method highlights domain-specific patterns in WTP, it might not fully reflect variations in payment amounts. One study that quantitatively assessed variations in payment amounts found that WTP tended to increase with age and was also higher among individuals with chronic conditions, which aligns with our findings [34]. Building on this, future research should use more detailed measurement methods that can capture not only whether individuals are willing to pay but also how much they are willing to pay, allowing for a better understanding of payment preferences for telemedicine and teleconsultation services.

Third, the survey was self-reported; thus, measurement errors are possible. Our reliance on self-reported data means there was an inherent limitation regarding the accuracy of income responses; this led us to exclude income as a covariate. Although our findings indicated that WTP tended to increase with age, it is highly likely that increased age is also associated with greater accumulated wealth and higher income. To address this, we conducted supplementary analyses incorporating income as a covariate to examine WTP for telemedicine and teleconsultation (Table S8 in Multimedia Appendix 1). These additional analyses revealed trends similar to our main results, confirming that WTP remained higher among older age groups even after adjusting for income. In addition, limitations regarding the absence of clinical data such as blood glucose levels, numerical values, and blood pressure existed. However, efforts have been made to minimize these limitations using a reliable, large web-based panel with approximately 70,000 registered panelists that could represent South Korea’s population.

This study examined key factors influencing the willingness to use and pay for telemedicine and teleconsultation across 5 clinical domains in South Korea. The findings showed that user acceptance differed depending on prior telemedicine experience, age, health status, and the type of clinical service.

These results suggest that telehealth strategies should be tailored to the characteristics of different user groups and clinical needs. Rather than applying a single standardized model, more flexible and responsive approaches may be needed to accommodate the diverse expectations and conditions associated with each domain.

By identifying patterns of acceptance using a nationally representative sample, this study provides meaningful insights that can inform the development of inclusive, patient-centered telehealth policies. As digital health care continues to evolve, understanding user perspectives will remain essential for designing equitable and sustainable service models that address both medical priorities and population diversity.

## Appendix

Multimedia Appendix 1 Supplementary data.

## Abbreviations

OR: odds ratio
WTP: willingness to pay
WTU: willingness to use

## Data Availability

The datasets used and/or analyzed during this study are available from the corresponding author on reasonable request.
