# Supplementary material for: Willingness to Use and Pay for Telemedicine and Teleconsultation Across Five Clinical Domains in South Korea: Cross-Sectional Survey
Source: J Med Internet Res. 2025 Jun 16;27:e65304. doi: 10.2196/65304 (PMC12209718; doi:10.2196/65304)
Supplement: Multimedia Appendix 1 [file jmir_v27i1e65304_app1.pdf]

Table S1. The changes in South Korea's telemedicine policy.

Date	Highlights	Comments
**February, 2020**	Telephone consultation/prescription and proxy prescription was temporary allowed (Feb 24, 2020.) as the National crisis alert level against infectious diseases was raised to "Red(Level IV)" (Feb 23, 2020.)	-
**December, 2020**	By the amendment of the Infectious Disease Control and Prevention Act, temporary telephone consultation/prescription and proxy prescription was institutionalized (Dec 15, 2020.)	1. Temporary telephone consultation/prescription and proxy prescription was terminated as the National crisis alter level against infectious diseases was lowered to "Orange(Level III)" (Jun 01, 2023.)<br><br>2. Temporary telephone consultation/prescription and proxy prescription: 37.86 million cases for 14.19 million people were conducted (Feb 24, 2020. ~ Apr 30, 2023. Health Insurance Review & Assessment Service.)
**June, 2023**	Jun 01, 2023., Implementing the pilot project of telemedicine for limited patients by Ministry of Health and Welfare	Grace period: Jun 01, 2023. ~ Aug 31, 2023.
**September, 2023**	Announcement of the Amendment to the Guidelines for pilot projects of telemedicine	Announcement of the Amendment as the grace period ends on Aug 31, 2023.
**December, 2023**	Dec 06, 2023., Announcement of the Amendment to the Guidelines for pilot projects of telemedicine (Date of enforcement: Dec 15, 2023. ~)	Significantly easing the eligibility requirements for the project subjects(patients)

Figure S1. Density of general hospitals by area.

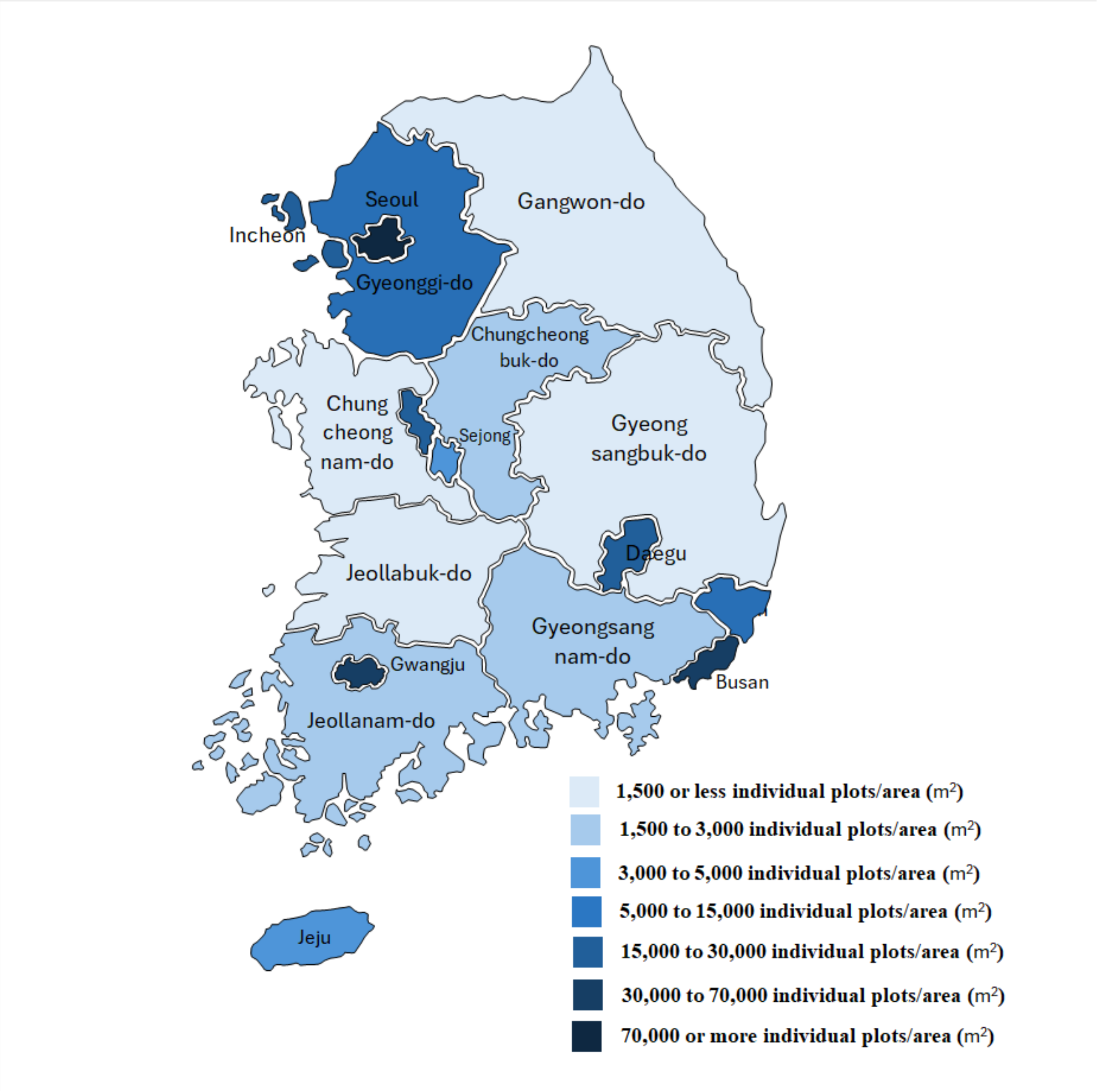

Table S2. General characteristics of the study population.

	Total		Willing to Use Telemedicine					Willing to Pay Telemedicine					Willing to Use Teleconsultation					Willing to Pay Teleconsultation				
			Yes		No			Yes		No			Yes		No			Yes		No		
	N	%	N	%	N	%	*P* value	N	%	N	%	*P* value	N	%	N	%	*P* value	N	%	N	%	*P* value
**Demographics**	552	100.00	445	100.00	107	100.00		242	100.00	310	100.00		429	100.00	123	100.00		227	100	325	100.00	
**Sex**																						
Female	289	52.36	230	79.58	59	20.42	.52	130	44.98	159	55.02	.57	228	78.89	61	21.11	.49	122	42.21	167	57.79	.59
Male	263	47.64	215	81.75	48	18.25		112	42.59	151	57.41		201	76.43	62	23.57		105	39.92	158	60.08	
**Age group**																						
19~29	87	15.76	74	85.06	13	14.94		31	35.63	56	64.37		60	68.97	27	31.03		22	25.29	65	74.71	
30~39	101	18.30	80	79.21	21	20.79		33	32.67	68	67.33		75	74.26	26	25.74		37	36.63	64	63.37	
40~49	118	21.38	95	80.51	23	19.49	.23	59	50.00	59	50.00	.001	92	77.97	26	22.03	.09	50	42.37	68	57.63	<.001
50~59	136	24.64	102	75.00	34	25.00		56	41.18	80	58.82		109	80.15	27	19.85		56	41.18	80	58.82	
60+	110	19.93	94	85.45	16	14.55		63	57.27	47	42.73		93	84.55	17	15.45		62	56.36	48	43.64	
**Residence**																						
Seoul Capital Area	284	51.45	232	81.69	52	18.31	.51	131	46.13	153	53.87	.27	213	75.00	71	25.00	.11	118	41.55	166	58.45	.83
Others	268	48.55	213	79.48	55	20.52		111	41.42	157	58.58		216	80.60	52	19.40		109	40.67	159	59.33	
**Alcohol use**																						
No	107	19.38	88	82.24	19	17.76	.64	45	42.06	62	57.94	.68	86	80.37	21	19.63	.46	45	42.06	62	57.94	.83
Yes	445	80.62	357	80.22	88	19.78		197	44.27	248	55.73		343	77.08	102	22.92		182	40.90	263	59.10	
**Smoking**																						
Non-smoker	255	46.20	201	78.82	54	21.18		109	42.75	146	57.25		188	73.73	67	26.27		66	25.88	189	74.12	
Ex-smoker	134	24.28	117	87.31	17	12.69	.08	61	45.52	73	54.48	.87	115	85.82	19	14.18	.02	59	44.03	75	55.97	.73
Current smoker	163	29.53	127	77.91	36	22.09		72	44.17	91	55.83		126	77.30	37	22.70		102	62.58	61	37.42	
**Environment**																						

**Distance to healthcare**																						
Less than 5 minutes on foot	78	14.13	67	85.90	11	14.10		41	52.56	37	47.44		64	82.05	14	17.95		38	48.72	40	51.28	
5 to 9 minutes on foot	191	34.60	155	81.15	36	18.85	.55	85	44.50	106	55.50	.24	149	78.01	42	21.99	.68	84	43.98	107	56.02	.20
10 to 29 minutes on foot	199	36.05	156	78.39	43	21.61		85	42.71	114	57.29		150	75.38	49	24.62		72	36.18	127	63.82	
30 minutes or more on foot	84	15.22	67	79.76	17	20.24		31	36.90	53	63.10		66	78.57	18	21.43		33	39.29	51	60.71	
**Subjective distance to healthcare**																						
Very close	114	20.65	92	80.70	22	19.30		57	50.00	57	50.00		93	81.58	21	18.42		55	48.25	59	51.75	
Close	342	61.96	279	81.58	63	18.42	.62	152	44.44	190	55.56	.07	263	76.90	79	23.10	.53	140	40.94	202	59.06	.09
Far	96	17.39	74	77.08	22	22.92		33	34.38	63	65.63		73	76.04	23	23.96		32	33.33	64	66.67	
**Service Experience**																						
**Experiences of telemedicine**																						
No	474	85.87	372	78.48	102	21.52	.002	191	40.30	283	59.70	<.001	361	76.16	113	23.84	.03	179	37.76	295	62.24	<.001
Yes	78	14.13	73	93.59	5	6.41		51	65.38	27	34.62		68	87.18	10	12.82		48	61.54	30	38.46	
**Health Status**																						
**SRH**																						
Bad	377	68.30	300	79.58	77	20.42	.36	156	41.38	221	58.62	.09	287	76.13	90	23.87	.19	148	39.26	229	60.74	.19
Good	175	31.70	145	82.86	30	17.14		86	49.14	89	50.86		142	81.14	33	18.86		79	45.14	96	54.86	
**Experiences of Chronic disease**																						
No	280	50.72	224	80.00	56	20.00	.71	100	35.71	180	64.29	<.001	207	73.93	73	26.07	.03	94	33.57	186	66.43	<.001
Yes	272	49.28	221	81.25	51	18.75		142	52.21	130	47.79		222	81.62	50	18.38		133	48.90	139	51.10	
**Experiences of Mental disease**																						
No	474	85.87	383	80.80	91	19.20	.79	204	43.04	270	56.96	.35	369	77.85	105	22.15	.86	192	40.51	282	59.49	.47
Yes	78	14.13	62	79.49	16	20.51		38	48.72	40	51.28		60	76.92	18	23.08		35	44.87	43	55.13	

Table S3. Results of factors associated with willingness to use and pay for telemedicine and teleconsultation by musculoskeletal disorders.

	Willing to Use Telemedicine				Willing to Pay Telemedicine				Willing to Use Teleconsultation				Willing to Pay Teleconsultation			
	OR[a]	90% CI[b]		*P* value	OR	90% CI		*P* value	OR	90% CI		*P* value	OR	90% CI		*P* value
**Demographics**																
**Sex**																
Female	Ref[c]				Ref				Ref				Ref			
Male	0.91	0.65	1.28	.64	0.68	0.44	1.07	.16	0.86	0.61	1.21	.46	0.63	0.42	0.93	.05
**Age group**																
19~29	Ref				Ref				Ref				Ref			
30~39	0.59	0.35	1.00	.001	0.48	0.22	1.02	.06	0.96	0.58	1.58	.19	1.29	0.68	2.43	.73
40~49	1.04	0.63	1.72	.57	0.85	0.43	1.66	.99	0.98	0.60	1.62	.22	1.12	0.59	2.11	.31
50~59	1.63	1.00	2.66	.04	0.76	0.39	1.47	.63	1.73	1.06	2.83	.04	1.49	0.81	2.73	.73
60+	1.97	1.16	3.35	.006	1.44	0.74	2.82	.03	1.64	0.97	2.79	.13	2.42	1.30	4.52	.008
**Residence**																
Seoul Capital Area	Ref				Ref				Ref				Ref			
Non-capital	1.41	1.05	1.91	.06	1.34	0.90	2.00	.23	1.34	1.00	1.81	.10	1.58	1.12	2.23	.03
**Alcohol use**																
No	Ref				Ref				Ref				Ref			
Yes	1.20	0.80	1.79	.45	0.77	0.44	1.36	.45	1.21	0.81	1.80	.43	0.92	0.58	1.47	.77
**Smoking**																
Non-smoker	Ref				Ref				Ref				Ref			
Ex-smoker	1.29	0.85	1.94	.61	1.41	0.81	2.44	.85	1.29	0.86	1.94	.54	1.03	0.64	1.66	.42
Current smoker	1.32	0.89	1.97	.47	1.79	1.06	3.02	.14	1.27	0.86	1.89	.58	1.59	1.01	2.50	.06
**Environment**																
**Distance to healthcare**																
Less than 5 minutes on foot	Ref				Ref				Ref				Ref			

5 to 9 minutes on foot	0.85	0.51	1.44	.90	0.73	0.39	1.37	.88	0.86	0.51	1.44	.66	1.39	0.77	2.50	.42
10 to 29 minutes on foot	0.77	0.44	1.35	.60	0.63	0.31	1.28	.60	0.77	0.44	1.35	.82	0.99	0.52	1.91	.33
30 minutes or more on foot	0.75	0.35	1.61	.68	0.54	0.20	1.48	.48	0.61	0.29	1.30	.33	1.49	0.62	3.58	.49
**Subjective distance to healthcare**																
Very close	Ref				Ref				Ref				Ref			
Close	1.07	0.68	1.68	.40	0.64	0.36	1.13	.26	1.12	0.71	1.75	.55	0.71	0.43	1.18	.95
Far	0.78	0.39	1.58	.44	0.81	0.32	2.03	.99	0.96	0.49	1.91	.79	0.53	0.23	1.19	.27
**Service Experience**																
**Experiences of telemedicine**																
No	Ref				Ref				Ref				Ref			
Yes	1.34	0.87	2.06	.27	2.27	1.38	3.75	.007	1.41	0.91	2.17	.19	2.17	1.38	3.41	.005
**Health Status**																
**Self-rated health**																
Bad	Ref				Ref				Ref				Ref			
Good	1.52	1.09	2.13	.04	1.34	0.86	2.07	.28	1.12	0.80	1.56	.57	1.26	0.86	1.85	.33
**Experiences of Chronic disease**																
No	Ref				Ref				Ref				Ref			
Yes	1.33	0.96	1.85	.15	1.43	0.92	2.23	.19	1.05	0.76	1.45	.81	1.35	0.93	1.98	.19
**Experiences of Mental disease**																
No	Ref				Ref				Ref				Ref			
Yes	1.35	0.87	2.09	.27	1.94	1.15	3.27	.04	0.92	0.60	1.43	.77	1.13	0.69	1.84	.68

[a] OR: Odds Ratio
[b] CI: Confidence Interval
[c] Reference values

Table S4. Results of factors associated with willingness to use and pay for telemedicine and teleconsultation by internal medicine disorders.

	Willing to Use Telemedicine				Willing to Pay Telemedicine				Willing to Use Teleconsultation				Willing to Pay Teleconsultation			
	OR[a]	90% CI[b]		*P* value	OR	90% CI		*P* value	OR	90% CI		*P* value	OR	90% CI		*P* value
**Demographics**																
**Sex**																
Female	Ref[c]				Ref				Ref				Ref			
Male	0.71	0.50	1.00	.10	0.84	0.55	1.29	.50	0.78	0.55	1.10	.24	0.66	0.45	0.97	.08
**Age group**																
19~29	Ref				Ref				Ref				Ref			
30~39	0.95	0.57	1.57	.04	1.07	0.53	2.19	.45	1.42	0.86	2.35	.36	1.49	0.79	2.80	.78
40~49	1.39	0.84	2.30	.99	1.31	0.66	2.61	.96	1.73	1.04	2.86	.91	1.20	0.64	2.26	.46
50~59	1.90	1.16	3.10	.07	1.15	0.58	2.27	.58	2.14	1.30	3.52	.17	1.40	0.76	2.59	.998
60+	2.11	1.24	3.60	.04	2.28	1.14	4.54	.01	2.62	1.51	4.53	.03	2.15	1.14	4.06	.04
**Residence**																
Seoul Capital Area	Ref				Ref				Ref				Ref			
Non-capital	1.44	1.06	1.94	.047	1.09	0.75	1.59	.71	1.33	0.98	1.81	.12	1.43	1.02	2.01	.08
**Alcohol use**																
No	Ref				Ref				Ref				Ref			
Yes	1.16	0.78	1.72	.55	0.97	0.58	1.62	.92	1.23	0.82	1.86	.41	0.87	0.55	1.37	.61
**Smoking**																
Non-smoker	Ref				Ref				Ref				Ref			
Ex-smoker	1.56	1.03	2.36	.42	1.12	0.67	1.89	.68	1.17	0.77	1.78	.93	1.54	0.98	2.44	.39
Current smoker	1.71	1.15	2.54	.14	1.58	0.96	2.61	.13	1.31	0.88	1.96	.37	1.58	1.00	2.49	.31
**Environment**																
**Distance to healthcare**																
Less than 5 minutes on foot	Ref				Ref				Ref				Ref			

5 to 9 minutes on foot	0.98	0.58	1.66	0.86	0.87	0.48	1.58	0.19	0.79	0.46	1.36	0.63	1.22	0.69	2.16	0.68
10 to 29 minutes on foot	1.09	0.62	1.91	0.64	0.52	0.27	1.03	0.25	0.84	0.47	1.50	0.89	0.97	0.51	1.81	0.39
30 minutes or more on foot	0.97	0.46	2.08	0.89	0.43	0.16	1.13	0.23	0.81	0.38	1.76	0.85	1.39	0.59	3.29	0.51
**Subjective distance to healthcare**																
Very close	Ref				Ref				Ref				Ref			
Close	0.73	0.46	1.16	0.75	0.68	0.40	1.15	0.17	0.83	0.52	1.32	0.89	0.59	0.36	0.95	0.59
Far	0.62	0.31	1.25	0.37	0.98	0.41	2.37	0.70	0.64	0.32	1.30	0.33	0.45	0.20	1.01	0.21
**Service Experience**																
**Experiences of telemedicine**																
No	Ref				Ref				Ref				Ref			
Yes	1.19	0.77	1.83	0.51	2.71	1.69	4.34	0.001	0.99	0.64	1.53	0.97	1.68	1.07	2.65	0.06
**Health Status**																
**Self-rated health**																
Bad	Ref				Ref				Ref				Ref			
Good	1.12	0.80	1.56	0.59	0.98	0.64	1.50	0.94	1.24	0.88	1.74	0.31	0.89	0.60	1.31	0.61
**Experiences of Chronic disease**																
No	Ref				Ref				Ref				Ref			
Yes	1.33	0.96	1.84	0.15	1.43	0.94	2.18	0.16	0.98	0.71	1.37	0.94	1.25	0.86	1.81	0.33
**Experiences of Mental disease**																
No	Ref				Ref				Ref				Ref			
Yes	0.99	0.64	1.54	0.96	1.48	0.89	2.46	0.20	0.87	0.56	1.36	0.61	1.09	0.67	1.77	0.78

[a] OR: Odds Ratio
[b] CI: Confidence Interval
[c] Reference values

Table S5. Results of factors associated with willingness to use and pay for telemedicine and teleconsultation by dermatological disorders.

	Willing to Use Telemedicine				Willing to Pay Telemedicine				Willing to Use Teleconsultation				Willing to Pay Teleconsultation			
	**OR[a]**	**90% CI[b]**		***P* value**	**OR**	**90% CI**		***P* value**	**OR**	**90% CI**		***P* value**	**OR**	**90% CI**		***P* value**
**Demographics**																
**Sex**																
Female	Ref[c]				Ref				Ref				Ref			
Male	1.39	0.99	1.95	.12	1.17	0.76	1.80	.54	1.08	0.76	1.53	.71	1.19	0.80	1.77	.48
**Age group**																
19~29	Ref				Ref				Ref				Ref			
30~39	0.58	0.34	0.96	.10	0.62	0.32	1.19	.39	1.50	0.89	2.51	.499	1.21	0.64	2.28	.91
40~49	0.79	0.47	1.32	.93	0.92	0.50	1.71	.39	1.32	0.79	2.19	.98	1.22	0.65	2.28	.94
50~59	0.74	0.45	1.23	.78	0.56	0.30	1.06	.17	1.18	0.72	1.94	.55	1.22	0.66	2.26	.96
60+	0.85	0.49	1.46	.66	0.83	0.43	1.61	.74	1.66	0.97	2.86	.24	1.61	0.85	3.05	.23
**Residence**																
Seoul Capital Area	Ref				Ref				Ref				Ref			
Non-capital	1.17	0.87	1.58	.38	1.20	0.82	1.74	.43	1.41	1.04	1.91	.07	1.47	1.04	2.09	.07
**Alcohol use**																
No	Ref				Ref				Ref				Ref			
Yes	1.49	1.00	2.23	.10	1.14	0.69	1.89	.67	1.30	0.86	1.97	.29	1.16	0.73	1.85	.61
**Smoking**																
Non-smoker	Ref				Ref				Ref				Ref			
Ex-smoker	1.14	0.76	1.73	.76	1.28	0.77	2.12	.40	1.80	1.16	2.77	.03	1.26	0.78	2.03	.78
Current smoker	1.14	0.77	1.69	.76	1.05	0.63	1.74	.78	1.18	0.79	1.76	.56	1.39	0.87	2.22	.38
**Environment**																
**Distance to healthcare**																
Less than 5 minutes on foot	Ref				Ref				Ref				Ref			

5 to 9 minutes on foot	0.81	0.48	1.37	.74	1.06	0.57	1.94	.29	0.80	0.47	1.38	.98	1.22	0.68	2.17	.23
10 to 29 minutes on foot	0.79	0.44	1.39	.61	0.71	0.36	1.40	.36	0.80	0.44	1.42	.92	0.81	0.43	1.56	.37
30 minutes or more on foot	0.83	0.39	1.78	.93	0.71	0.27	1.85	.61	0.66	0.31	1.44	.47	0.88	0.36	2.16	.78
**Subjective distance to healthcare**																
Very close	Ref				Ref		0.62		Ref				Ref			
Close	0.88	0.56	1.39	.99	0.78	0.46	1.34	.67	0.95	0.59	1.51	.78	0.64	0.39	1.05	.38
Far	0.78	0.39	1.56	.60	0.78	0.32	1.88	.78	0.79	0.39	1.60	.56	0.65	0.29	1.47	.63
**Service Experience**																
**Experiences of telemedicine**																
No	Ref				Ref				Ref				Ref			
Yes	1.44	0.93	2.23	.17	2.95	1.86	4.69	<.001	1.84	1.15	2.94	.03	1.75	1.09	2.79	.05
**Health Status**																
**Self-rated health**																
Bad	Ref				Ref				Ref				Ref			
Good	1.27	0.91	1.78	.24	0.93	0.61	1.42	.78	1.11	0.79	1.57	.61	1.12	0.76	1.65	.64
**Experiences of Chronic disease**																
No	Ref				Ref				Ref				Ref			
Yes	1.24	0.89	1.71	.29	1.16	0.77	1.76	.55	1.00	0.72	1.39	.99	1.22	0.83	1.79	.39
**Experiences of Mental disease**																
No	Ref				Ref				Ref				Ref			
Yes	1.29	0.83	2.01	.35	1.16	0.68	1.97	.65	0.81	0.52	1.25	.42	1.11	0.67	1.83	.74

[a] OR: Odds Ratio
[b] CI: Confidence Interval
[c] Reference values

Table S6. Results of factors associated with willingness to use and pay for telemedicine and teleconsultation by cancer disorders.

	Willing to Use Telemedicine				Willing to Pay Telemedicine				Willing to Use Teleconsultation				Willing to Pay Teleconsultation			
	OR[a]	90% CI[b]		*P* value	OR	90% CI		*P* value	OR	90% CI		*P* value	OR	90% CI		*P* value
**Demographics**																
**Sex**																
Female	Ref[c]				Ref				Ref				Ref			
Male	0.80	0.55	1.17	.33	0.84	0.53	1.34	.54	0.74	0.53	1.04	.15	0.74	0.50	1.09	.20
**Age group**																
19~29	Ref				Ref				Ref				Ref			
30~39	0.54	0.28	1.02	.002	0.42	0.19	0.95	.02	0.84	0.50	1.41	.02	1.02	0.54	1.91	.35
40~49	1.53	0.87	2.69	.17	0.70	0.34	1.44	.27	1.16	0.70	1.92	.50	1.13	0.61	2.09	.62
50~59	1.49	0.86	2.59	.19	1.11	0.57	2.18	.46	2.10	1.28	3.44	.005	1.29	0.71	2.34	.89
60+	1.78	0.99	3.20	.04	2.21	1.10	4.46	.001	1.87	1.10	3.18	.06	2.09	1.12	3.89	.02
**Residence**																
Seoul Capital Area	Ref				Ref				Ref				Ref			
Non-capital	1.25	0.90	1.74	.27	1.05	0.69	1.59	.85	1.63	1.21	2.19	.007	1.60	1.14	2.25	.02
**Alcohol use**																
No	Ref				Ref				Ref				Ref			
Yes	1.37	0.89	2.13	.23	0.50	0.27	0.94	.07	1.11	0.75	1.65	.66	0.72	0.45	1.16	.26
**Smoking**																
Non-smoker	Ref				Ref				Ref				Ref			
Ex-smoker	1.55	0.98	2.44	.74	1.47	0.82	2.63	.57	1.18	0.78	1.77	.93	1.17	0.73	1.88	.87
Current smoker	2.04	1.32	3.17	.03	1.55	0.89	2.68	.39	1.44	0.97	2.14	.18	1.50	0.95	2.35	.17
**Environment**																
**Distance to healthcare**																
Less than 5 minutes on foot	Ref				Ref				Ref				Ref			

5 to 9 minutes on foot	0.93	0.53	1.63	.55	0.75	0.37	1.49	.33	0.77	0.46	1.28	.45	1.64	0.90	3.01	.60
10 to 29 minutes on foot	0.99	0.54	1.83	.31	0.57	0.27	1.24	.86	0.83	0.47	1.44	.77	1.52	0.78	2.94	.93
30 minutes or more on foot	0.52	0.22	1.24	.14	0.30	0.10	0.86	.07	0.89	0.42	1.90	.92	1.99	0.82	4.79	.36
**Subjective distance to healthcare**																
Very close	Ref				Ref				Ref				Ref			
Close	0.84	0.51	1.36	.25	0.96	0.51	1.80	.04	0.92	0.59	1.44	.61	0.59	0.36	0.98	.69
Far	1.24	0.57	2.68	.45	3.09	1.20	7.93	.02	0.67	0.34	1.35	.32	0.43	0.20	0.97	.17
**Service Experience**																
**Experiences of telemedicine**																
No	Ref				Ref				Ref				Ref			
Yes	1.64	1.04	2.59	.08	1.81	1.06	3.11	.07	1.04	0.68	1.58	.90	1.87	1.19	2.95	.02
**Health Status**																
**Self-rated health**																
Bad	Ref				Ref				Ref				Ref			
Good	1.36	0.94	1.96	.17	1.05	0.66	1.66	.86	1.35	0.97	1.88	.14	0.79	0.54	1.17	.32
**Experiences of Chronic disease**																
No	Ref				Ref				Ref				Ref			
Yes	0.85	0.59	1.22	.46	0.82	0.51	1.31	.48	0.73	0.53	1.01	.11	0.91	0.62	1.32	.67
**Experiences of Mental disease**																
No	Ref				Ref				Ref				Ref			
Yes	1.58	1.00	2.52	.10	1.90	1.10	3.29	.05	0.88	0.57	1.36	.62	1.41	0.87	2.27	.24

[a] OR: Odds Ratio
[b] CI: Confidence Interval
[c] Reference values

Table S7. Results of factors associated with willingness to use and pay for telemedicine and teleconsultation by psychiatric disorders.

	Willing to Use Telemedicine				Willing to Pay Telemedicine				Willing to Use Teleconsultation				Willing to Pay Teleconsultation			
	OR[a]	90% CI[b]		*P* value	OR	90% CI		*P* value	OR	90% CI		*P* value	OR	90% CI		*P* value
**Demographics**																
**Sex**																
Female	Ref[c]				Ref				Ref				Ref			
Male	0.71	0.50	1.01	.11	0.75	0.51	1.10	.21	0.65	0.47	0.92	.04	0.84	0.58	1.23	.45
**Age group**																
19~29	Ref				Ref				Ref				Ref			
30~39	0.63	0.37	1.07	.67	0.82	0.46	1.44	.91	0.71	0.43	1.19	.20	0.64	0.36	1.14	.41
40~49	0.68	0.40	1.17	1.00	0.72	0.41	1.26	.59	0.80	0.48	1.33	.48	0.66	0.38	1.16	.46
50~59	0.48	0.29	0.80	.04	0.65	0.38	1.13	.29	0.95	0.58	1.55	.82	0.64	0.37	1.10	.33
60+	0.72	0.41	1.26	.81	0.85	0.48	1.51	.77	1.15	0.67	1.97	.24	0.99	0.56	1.75	.23
**Residence**																
Seoul Capital Area	Ref				Ref				Ref				Ref			
Non-capital	0.83	0.61	1.12	.31	1.02	0.73	1.42	.93	0.85	0.63	1.14	.36	1.16	0.83	1.62	.46
**Alcohol use**																
No	Ref				Ref				Ref				Ref			
Yes	0.89	0.60	1.34	.64	0.74	0.47	1.18	.29	0.92	0.62	1.37	.74	0.65	0.41	1.04	.13
**Smoking**																
Non-smoker	Ref				Ref				Ref				Ref			
Ex-smoker	1.07	0.70	1.63	.98	1.03	0.65	1.63	.86	1.22	0.81	1.84	.76	1.28	0.82	2.01	.29
Current smoker	1.13	0.75	1.70	.68	0.98	0.63	1.52	.87	1.30	0.87	1.93	.45	0.99	0.64	1.55	.58
**Environment**																
**Distance to healthcare**																
Less than 5 minutes on foot	Ref				Ref				Ref				Ref			

5 to 9 minutes on foot	0.92	0.54	1.58	.73	0.95	0.54	1.66	.52	1.43	0.84	2.43	.34	1.51	0.86	2.66	.49
10 to 29 minutes on foot	0.92	0.52	1.65	.71	0.75	0.40	1.38	.50	1.07	0.61	1.89	.42	1.15	0.62	2.15	.41
30 minutes or more on foot	0.67	0.31	1.44	.34	0.71	0.31	1.66	.59	1.44	0.67	3.08	.54	1.83	0.79	4.23	.30
**Subjective distance to healthcare**																
Very close	Ref				Ref				Ref				Ref			
Close	1.10	0.70	1.75	.51	1.19	0.73	1.96	.38	0.71	0.45	1.13	.93	0.69	0.43	1.12	.92
Far	0.90	0.45	1.82	.67	0.91	0.41	2.01	.66	0.48	0.24	0.97	.12	0.45	0.21	0.99	.14
**Service Experience**																
**Experiences of telemedicine**																
No	Ref				Ref				Ref				Ref			
Yes	1.19	0.76	1.87	.52	2.34	1.51	3.61	.001	1.57	1.00	2.46	.098	1.86	1.19	2.88	.02
**Health Status**																
**Self-rated health**																
Bad	Ref				Ref				Ref				Ref			
Good	0.98	0.70	1.38	.94	1.47	1.02	2.11	.09	0.85	0.61	1.18	.41	1.07	0.74	1.54	.77
**Experiences of Chronic disease**																
No	Ref				Ref				Ref				Ref			
Yes	1.43	1.02	1.99	.08	1.43	0.99	2.07	.11	1.17	0.85	1.62	.42	1.19	0.83	1.72	.42
**Experiences of Mental disease**																
No	Ref				Ref				Ref				Ref			
Yes	1.23	0.78	1.96	.46	1.49	0.94	2.36	.16	0.79	0.51	1.22	.36	1.55	0.98	2.46	.12

[a] OR: Odds Ratio
[b] CI: Confidence Interval
[c] Reference values

Table S8. Results of factors associated with willingness to pay for telemedicine and teleconsultation adjusted for Income Level.

	Willing to Pay Telemedicine				Willing to Pay Teleconsultation			
	OR[a]	90% CI[b]		*P* value	OR	90% CI		*P* value
**Demographics**								
**Sex**								
Female	Ref[c]				Ref			
Male	0.88	0.62	1.26	.56	0.88	0.62	1.26	.57
**Age group**								
19~29	Ref				Ref			
30~39	0.81	0.46	1.41	.06	1.62	0.92	2.88	.65
40~49	1.50	0.88	2.57	.21	1.89	1.08	3.32	.74
50~59	1.05	0.62	1.78	.48	1.82	1.06	3.15	.88
60+	1.88	1.08	3.27	.02	3.16	1.77	5.62	.003
**Residence**								
Seoul Capital Area	Ref				Ref			
Non-capital	0.99	0.73	1.35	.95	1.10	0.80	1.50	.63
**Alcohol use**								
No	Ref				Ref			
Yes	1.06	0.70	1.61	.81	1.18	0.78	1.79	.52
**Smoking**								
Non-smoker	Ref				Ref			
Ex-smoker	0.90	0.59	1.38	.61	0.93	0.61	1.42	.67
Current smoker	1.03	0.68	1.55	.73	1.04	0.69	1.58	.72
**Income**								
Q1 (lowest)	Ref				Ref			
Q2	1.77	1.10	2.84	.51	1.30	0.81	2.08	.61
Q3	2.01	1.23	3.28	.14	1.42	0.88	2.31	.28
Q4 (highest)	1.83	1.10	3.05	.41	1.13	0.68	1.87	.71
**Environment**								
**Distance to healthcare**								
Less than 5 minutes on foot	Ref				Ref			
5 to 9 minutes on foot	0.76	0.44	1.29	.70	0.83	0.49	1.41	.77
10 to 29 minutes on foot	0.69	0.39	1.24	.36	0.61	0.34	1.10	.04
30 minutes or more on foot	0.81	0.37	1.78	1.00	1.12	0.51	2.48	.37
**Subjective distance to healthcare**								
Very close	Ref				Ref			
Close	0.93	0.59	1.49	.52	0.88	0.55	1.40	.40
Far	0.65	0.31	1.35	.29	0.52	0.25	1.09	.13
**Service Experience**								
**Experiences of telemedicine**								

No	Ref				Ref			
Yes	2.78	1.77	4.38	<.001	2.72	1.74	4.25	<.001
**Health Status**								
**Self-rated health**								
Bad	Ref				Ref			
Good	1.63	1.15	2.31	.02	1.55	1.10	2.20	.04
**Experiences of Chronic disease**								
No	Ref				Ref			
Yes	1.89	1.35	2.65	.002	1.79	1.28	2.52	.005
**Experiences of Mental disease**								
No	Ref				Ref			
Yes	1.27	0.81	2.00	.38	1.19	0.76	1.88	.53

[a] OR: Odds Ratio
[b] CI: Confidence Interval
[c] Reference values
